# Microwave ablation plus camrelizumab monotherapy or combination therapy in non-small cell lung cancer

**DOI:** 10.3389/fonc.2022.938827

**Published:** 2022-08-26

**Authors:** Yahan Huang, Jiao Wang, Yanting Hu, Pikun Cao, Gang Wang, Hongchao Cai, Meixiang Wang, Xia Yang, Zhigang Wei, Xin Ye

**Affiliations:** ^1^ Department of Oncology, The First Affiliated Hospital of Shandong First Medical University & Shandong Provincial Qianfoshan Hospital, Shandong Lung Cancer Institute, Shandong Key Laboratory of Rheumatic Disease and Translational Medicine, Jinan, China; ^2^ Shandong First Medical University & Shandong Academy of Medical Sciences, Jinan, China; ^3^ Department of Oncology, Shandong Provincial Hospital Affiliated to Shandong First Medical University, Jinan, China; ^4^ Cheeloo College of Medicine, Shandong University, Jinan, China

**Keywords:** microwave ablation, camrelizumab, lung cancer, progression-free survival, overall survival, objective response rate

## Abstract

**Purpose:**

Immunotherapy has become widely applied in non-small cell lung cancer (NSCLC) patients. However, the relatively low response rate of immunotherapy monotherapy restricts its application. Combination therapy improves the response rate and prolongs patient survival; however, adverse events (AEs) associated with immunotherapies increase with combination therapy. Therefore, exploring combination regimens with equal efficacy and fewer AEs is urgently required. The aim of this study was to evaluate the efficacy and safety of microwave ablation (MWA) plus camrelizumab monotherapy or combination therapy in NSCLC.

**Materials and methods:**

Patients with pathologically confirmed, epidermal growth factor receptor/anaplastic lymphoma kinase-wild-type NSCLC were retrospectively enrolled in this study. Patients underwent MWA to the pulmonary lesions first, followed by camrelizumab monotherapy or combination therapy 5–7 days later. Camrelizumab was administered with the dose of 200 mg every 2 to 3 weeks. Treatment was continued until disease progression or intolerable toxicities. The technical success and technique efficacy of ablation, objective response rate (ORR), progression-free survival (PFS), overall survival (OS), complications of ablation, and AEs were recorded.

**Results:**

From January 1, 2019 to December 31, 2021, a total of 77 patients underwent MWA and camrelizumab monotherapy or combination therapy. Technical success was achieved in all patients (100%), and the technique efficacy was 97.4%. The ORR was 29.9%. The PFS and OS were 11.8 months (95% confidence interval, 9.5–14.1) and not reached, respectively. Smoking history and response to camrelizumab were correlated with PFS, and response to camrelizumab was correlated with OS in both the univariate and multivariate analyses. No periprocedural deaths due to ablation were observed. Complications were observed in 33 patients (42.9%). Major complications included pneumothorax (18.2%), pleural effusion (11.7%), pneumonia (5.2%), bronchopleural fistula (2.6%), and hemoptysis (1.3%). Grade 3 or higher AEs of camrelizumab, including reactive capillary endothelial proliferation, fatigue, pneumonia, edema, and fever, were observed in 10.4%, 6.5%, 5.2%, 2.6%, and 2.6% of patients, respectively.

**Conclusion:**

MWA combined with camrelizumab monotherapy or combination therapy is effective and safe for the treatment of NSCLC.

## 1 Introduction

Lung cancer is the leading cause of cancer-related mortality and morbidity in China and the second leading cause of cancer-related mortality worldwide (1, 2). Approximately 85% of lung cancers are non-small cell lung cancers (NSCLCs), of which adenocarcinoma and squamous cell carcinoma are the most common histological types (3). Most patients with lung cancer are diagnosed at an advanced stage, thus losing the opportunity for curative surgery. For these patients, the prognosis is quite poor, with a 5-year survival rate of only 0–10% (4).

The treatments for advanced NSCLC range from routine chemotherapy to novel targeted therapy and immunotherapy (5, 6). Compared with targeted therapy, which requires specific sensitive genetic mutations, immunotherapy targeting programmed death-1 (PD-1) or programmed death ligand-1(PD-L1) is more widely applied in NSCLC patients (7–9). However, the relatively low response rate of PD-1/PD-L1 antibody monotherapy restricts its application (7–9). The combination of PD-1/PD-L1 antibodies with other treatments including chemotherapy; anti-vascular endothelial growth factor receptor therapy plus chemotherapy; or another immunotherapy, mainly anti-cytotoxic lymphocyte antigen-4 antibody alone or in combination with chemotherapy, improves the response rate and prolongs survival (10–14). However, the adverse events (AEs) associated with immunotherapies increase with combination therapy (10–14). Therefore, exploring combination regimens with equal efficacy and fewer AEs is urgently required.

Accumulating evidence shows that microwave ablation (MWA) is an alternative treatment method for early-stage NSCLC patients with contraindications to surgery, such as cardiopulmonary insufficiency (15–17). Moreover, previous studies have verified that MWA plus chemotherapy or targeted therapy has a survival advantage over chemotherapy or targeted therapy alone (18–20). Our previous study showed that, for advanced NSCLC patients, the combination of MWA and camrelizumab (a PD-1 antibody designed by Hengrui Pharm, Jiangsu Province, China) improved the objective response rate (ORR) to 33.3%, which was higher than in previous reports where the ORR was around 20%. However, only 21 patients were included in the study, which limits the credibility of the results (21). Therefore, we conducted this retrospective study with a larger sample size to verify the efficacy and safety of MWA and camrelizumab monotherapy or combination therapy in advanced NSCLC.

## 2 Materials and methods

### 2.1 Patients

Patients meeting the following criteria were retrospectively enrolled: 1) pathologically or cytologically confirmed NSCLC; 2) advanced tumor stage, including stages III (unfit for radical surgery or irradiation) and IV, or recurrence post-local radical therapy (e.g., surgery, irradiation, or thermal ablation); 3) Eastern Cooperative Oncology Group performance status of 0 to 2; 4) one or more measurable lesions other than those for which MWA was performed; 5) tumors located in the peripheral lung; 6) wild-type epidermal growth factor receptor and anaplastic lymphoma kinase based on genetic test results; and 7) sufficient hepatic, renal, and cardiac function for MWA and anti-PD-1 treatment.

The exclusion criteria were as follows: 1) small cell lung cancer or neuroendocrine tumor in combination with NSCLC; 2) other malignant tumors during the previous 5 years; 3) active autoimmune disease requiring intervention; and 4) long-term administrations of hormones or anti-infective treatments within 2 weeks of MWA.

This study was conducted in accordance with the Declaration of Helsinki. The ethics committees of the First Affiliated Hospital of Shandong First Medical University and Shandong Provincial Hospital affiliated to Shandong First Medical University (SWYX: No.2019-004) approved this study. Written informed consent was obtained from all patients.

### 2.2 MWA procedure

All patients underwent preoperative analgesia. Local anesthesia was induced with 100 mg of lidocaine and 75 mg of bupivacaine. Sedation was induced with 10 mg of diazepam. The MWA procedure for lung tumors has been described in detail in our previous report (18). The operator briefly used computed tomography (CT) to locate the tumor before ablation, after which the antenna was inserted into the tumor step-by-step. CT was performed immediately after ablation to identify complications, such as pneumothorax and pleural effusion, and intervene if necessary. CT was also used to monitor the ablative response and range. The number of antennas was determined by the maximum transverse diameter of the tumor. When the tumor was 3 cm or larger, two antennas were used; otherwise, one antenna was used. The ablative zone was nearly 3.5 cm × 3 cm for MWA with the output was 60–80W/6–8 min. When the ground-glass opacity surpassed the tumor lesions by 5–10 mm, the ablation was terminated.

### 2.3 Camrelizumab administration

Anti-PD-1 treatment was administered 5–7 days after ablation. Camrelizumab was administered as a 30-minute intravenous infusion at a dose of 200 mg every 2 or 3 weeks until disease progression or unacceptable toxicity. For patients who received MWA plus camrelizumab as first-line treatment, camrelizumab was administered combined with chemotherapy, targeted therapy, or both. For those receiving this treatment as second- or later-line treatment, camrelizumab monotherapy was recommended. Chemotherapy regimens on a 21-day cycle, including pemetrexed (one dose of 500 mg/m^2^), nab-paclitaxel (one dose of 260 mg/m^2^), docetaxel (one dose of 75 mg/m^2^), and nedaplatin (80 mg/m^2^ over 2 days), were administered based on the physician’s preference. For targeted therapy, apatinib (250 mg once daily) or anlotinib (12 mg once daily) for 2 weeks on a 21-day cycle was administered continuously. Bevacizumab (7.5–15 mg/m^2^) was also administered once every 3 weeks to some patients.

### 2.4 Efficacy of MWA and camrelizumab

CT was conducted every 2 months during camrelizumab treatment and every 3 months thereafter.

Technical success and technique efficacy are commonly used to evaluate response to MWA. Technical success is a measure of whether the tumor was treated according to the protocol and covered completely by the ablation zone and was assessed during the procedure. Technique efficacy refers to a result of “complete ablation” of macroscopic tumors, as evidenced by follow-up imaging, at a prospectively defined time point (generally 1 month or later) (22).

The response to camrelizumab monotherapy or combination therapy was evaluated using the Response Evaluation Criteria in Solid Tumors version 1.1, which categorized responses as complete response (CR), partial response (PR), stable disease, or progressive disease (PD) (23). The ORR was defined as the proportion of patients who achieved CR and PR. The disease control rate was defined as the proportion of patients who did not show disease progression.

### 2.5 Safety of MWA and camrelizumab monotherapy or combination therapy

#### 2.5.1 Complications of MWA

The complications of MWA were divided into major complications and minor complications according to the Society of Intervention Response criteria (24). Major complications were defined as events that led to substantial morbidity and disability, which increase the level of care or lengthen the hospital stay. A blood transfusion or interventional drainage procedure is generally required for these patients. All other complications were considered minor (22).

#### 2.5.2 AEs of camrelizumab

The AEs of camrelizumab monotherapy or combination therapy were evaluated according to the Common Terminology Criteria of Adverse Events version 5.0 (25). Generally, when the severity of the AE reached or exceeded grade 3, camrelizumab treatment was paused, and intervention was conducted (22). Once the AEs were resolved, whether or not to resume camrelizumab treatment was decided on by the researchers. When disease progression occurred, patients could continue on camrelizumab until clinical symptom deterioration or PD confirmation in the imaging assessment.

### 2.6 Statistical analysis

SPSS software (version 17.0; SPSS Inc., Chicago, IL, USA) was used to perform the analyses. Numerical variables are described as means and standard deviations or medians and interquartile ranges according to distribution. Categorical variables are described as percentages. Progression-free survival (PFS) was defined as the time from MWA to the first documentation of PD or death. Overall survival (OS) was defined as the time from MWA to death. PFS and OS curves were estimated using the Kaplan–Meier method. The associations between PFS and OS and clinical characteristics were analyzed using univariate Cox regression and log-rank tests. Factors with *P* < 0.2 in the univariate analysis were included in the multivariate Cox proportional hazard model, which was applied to estimate hazard ratios and corresponding 95% confidence intervals. All statistical tests were two-sided, and statistical significance was defined as *P* < 0.05.

## 3 Results

### 3.1 Baseline characteristics

#### 3.1.1 Patients

**Figure 1:**
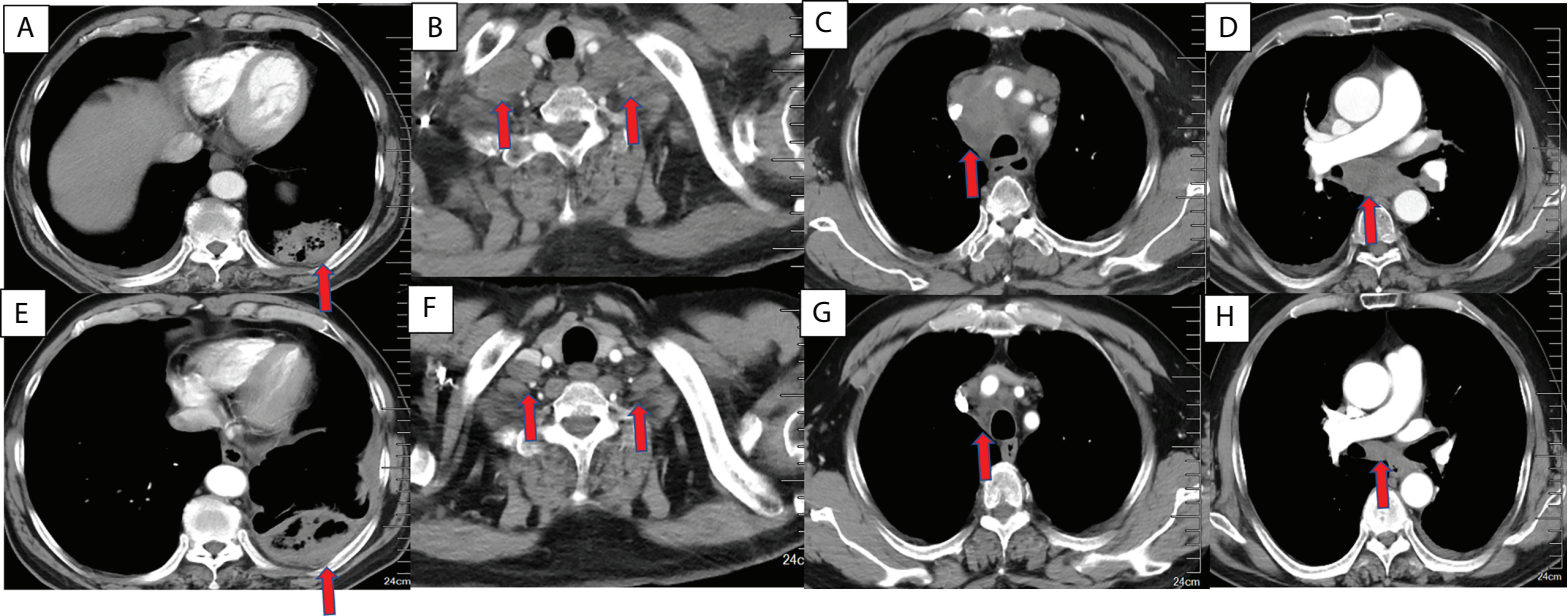
Chest CT findings of a 63-year-old male squamous cell lung cancer patient with a PD-L1 TPS of 1% who underwent MWA and camrelizumab monotherapy. **(A–D)** Baseline chest CT showing the primary tumor and lymph node metastases. **(A)** The primary tumor was located in the left lower lobe. **(B)** Bilateral supraclavicular lymph node metastases. **(C, D)** Mediastinal lymph node metastases in the 2R, 4R, 5, and 7 zones. **(E–H)** CT 2 months post-ablation and after four cycles of camrelizumab. **(E)**. Complete ablation was achieved in the primary tumor. **(F–H)**. Bilateral supraclavicular mediastinal lymph node metastases decreased dramatically. PD-L1, programmed death ligand-1; TPS, tumor proportion score; MWA, microwave ablation; CT, computed tomography.

**Figure 2:**
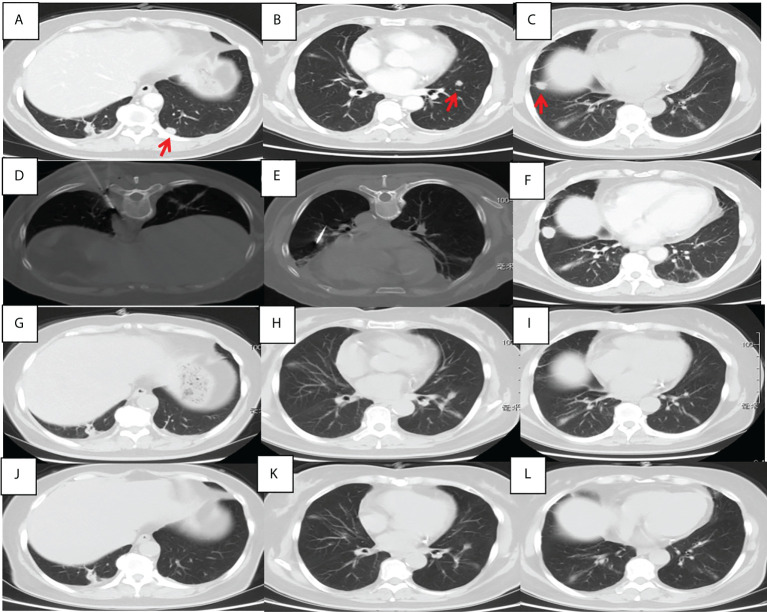
Chest CT findings of a 56-year-old female pulmonary adenocarcinoma patient with an unknown PD-L1 TPS who underwent MWA and camrelizumab monotherapy. **(A–C)** Baseline chest CT showing two metastases in the left lower lobe. **(D, E)** MWA was conducted for the tumor in the left lower lobe. **(F)** Two cycles of camrelizumab monotherapy were administered, after which the right metastasis enlarged. **(G–I)** Six months post-ablation, the left metastases achieved complete ablation and the right metastasis disappeared; complete response was achieved. **(J–L)** Nineteen months post-ablation, there was no indication of any pulmonary metastases. PD-L1, programmed death ligand-1; TPS, tumor proportion score; MWA, microwave ablation; CT, computed tomography.

From January 1, 2019 to December 31, 2021, A total of 132 patients underwent camrelizumab treatment were screened. Among them, 40 patients failed underwent MWA, 8 patients with stage IB to IIB and 4 patients with other cancers during the past 5 years. A total of 77 patients were retrospectively enrolled, with a mean age of 67.1 years (range, 48–82 years). Patient characteristics are shown in **Table 1**. Most patients were men (59, 76.6%), 65 years old or older (53, 68.8%), and smokers (53, 68.8%). Seventy-one patients had an Eastern Cooperative Oncology Group performance status of 1 (92.2%), and 60 had clinical stage IV disease (77.9%). Adenocarcinoma was the most common histological type (50, 64.9%). Most patients underwent MWA and camrelizumab monotherapy (45, 58.4%). For those who underwent camrelizumab combination therapy, a combination with chemotherapy (19 patients, 59.4%) was the most common, followed by targeted therapy (8 patients, 25.0%) and targeted therapy plus chemotherapy (5 patients, 15.6%). Two representative cases are shown in **Figures 1**, **2**.

**Table 1:** Baseline characteristics of the enrolled patients.

	N=77	Percent (%)
Gender		
Male	59	76.6
Female	18	23.4
Age		
Mean±SD(years old)	67.1±7.6	
<65 years old	24	31.2
≥65 years old	53	68.8
Smoking history		
Smokers	53	68.8
Non-smokers	24	31.2
ECOG		
0	4	5.2
1	71	92.2
2	2	2.6
Pathology		
Adenocarcinoma	50	64.9
Non-adenocarcinoma	27	35.1
Pathology		
Squamous cell lung cancer	22	28.6
Non-Squamous cell lung cancer	55	71.4
Stage		
III	17	22.1
IV	60	77.9
Lymph nodes metastases		
No	23	29.9
Yes	54	70.1
Distant metastases		
No	19	24.7
Yes	58	75.3
EGFR mutations		
Positive	9	11.7
Negative	26	33.8
Unknown	42	54.5
ALK mutations		
Positive	0	0.0
Negative	35	45.5
Unknown	42	54.5
PD-L1 expression		
Positive	11	14.3
Negative	4	5.2
Unknown	62	80.5
PD-L1 positive (TPS)		
1%-49%	5	6.5
≥50%	6	7.8

ALK, anaplastic lymphoma kinase; ECOG, Eastern Cooperation of Oncology Group; EGFR, epidermal growth factor receptor; N, number; PD-L1, programmed death-ligand-1; SD, standard deviation; TPS, total proportion score.

#### 3.1.2 Pulmonary tumors

All 77 pulmonary tumors were treated with MWA. The mean maximal transverse diameter was 3.3 centimeters, and 39 tumors (50.6%) had a diameter of 3.0 cm or larger. The right lung (40, 51.9%) and upper lobe (42, 54.5%) were the most common tumor sites. The output power of the antenna was generally fixed at 40 W, with a mean ablative time of 14.6 minutes. More information on tumor size and ablation is shown in **Table 2**.

**Table 2:** Baseline characteristics of the tumors underwent MWA.

	N=77	Percent(%)
Tumor size (cm)		
Mean(±SD)	3.3±2.2	
<3	38	49.4
≥3	39	50.6
Location of tumors		
Left lung	37	48.1
Right lung	40	51.9
Location of tumors		
Upper lobe	42	54.5
Middle and lower lobe	35	45.5
Location of tumors		
Upper right lobe	19	24.6
Middle right lobe	9	11.7
Lower right lobe	11	14.3
Upper left lobe	23	29.9
Lower left lobe	15	19.5
Number of antennas		
1	31	40.3
2	43	55.8
3	3	3.9
Power		
30W	14	18.2
40W	30	39.0
50W	18	23.4
60W	15	19.5
Ablation Duration (minutes)		
Mean±SD	14.6±9.1	

SD, standard deviation; W, watt.

### 3.2 Treatment efficacy

Although technical success was achieved in all patients (100%), the technique efficacy was 97.4% because two patients with tumors larger than 5 cm failed to achieve CR.

All patients underwent at least 1 cycle of camrelizumab treatment, and the median was 5 cycles (range, 1–53) (**Table 3**). Thirty-two patients (41.6%) received camrelizumab combination therapy. CR, PR, stable disease, and PD were achieved in 3, 20, 23, and 31 patients, respectively. The ORR and disease control rate were 29.9% and 59.7%, respectively.

**Table 3:** Camrelizumab treatments.

	N=77	Percent (%)
Camrelizumab therapy		
Combination	32	41.6
Monotherapy	45	58.4
Treatment Regimen		
First line	29	37.7
Subsequent line	48	62.3
Combination regimens(N=32)		
Targeted therapy	8	25.0
Chemotherapy	19	59.4
Targeted and chemotherapy	5	15.6
Treatment interval		
Two weeks	45	58.4
Three weeks	32	41.6
Treatment interruption		
Yes	13	16.9
No	64	83.1
Response		
CR	3	3.9
PR	20	25.9
SD	23	29.9
PD	31	40.3
ORR		
CR+PR	23	29.9
SD+PD	54	70.1
DCR		
CR+PR+SD	46	59.7
PD	31	40.3
Disease progression		
Yes	48	62.3
No	29	37.7

CR, complete response; ORR, objective response rate; DCR, disease control rate; PD, progression disease; PR, partial response; SD, stable disease; MWA, microwave ablation.

### 3.3 Survival analysis

**Figure 3:**
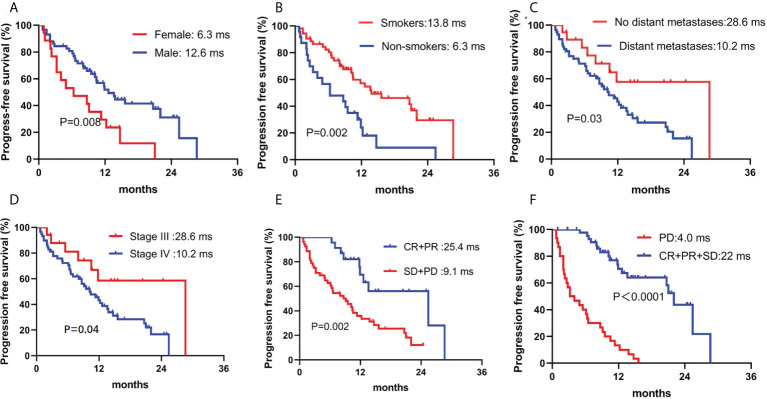
Correlation between PFS and **(A)** sex, **(B)** smoking history, **(C)** distant metastases, **(D)** stage, **(E)** ORR, and **(F)** DCR. PFS: progression-free survival; ORR, overall response rate; DCR, disease control rate.

Patients were followed through April 4, 2022, with a median follow-up period of 13.7 (4.3–36.2) months. Forty-eight patients (62.3%) had PD, and 26 patients (33.8%) died. The median PFS was 11.8 months (95% confidence interval, 9.5–14.1). However, the median OS was not reached. Sex, smoking history, stage, and response to camrelizumab were correlated with PFS in the univariate analysis. Age, smoking history, tumor size, and response to camrelizumab were correlated with PFS in the multivariate analysis (**Table 4**, **Figures 3A–D**). Smoking history and response to camrelizumab correlated with PFS in both the univariate and multivariate analyses (**Table 4**). Patients with smoking history and with CR or PR to camrelizumab had superior PFS compared to those without a smoking history (13.8 months vs. 6.3 months, *P* = 0.002) and those who failed to achieve CR or PR (25.4 months vs. 9.3 months, *P* = 0.003), respectively (**Figures 3E, F**).

**Table 4:** The univariate and multivariate analyses of progression free survival.

	Univariate analyses Multivariate analyses								
	Median PFS (95% CI)	HR (95% CI)	*P* value	β	S.E.	Wald	df	HR (95% CI)	*P* value
Gender		2.220 (1.045-4.715)	0.008	-0.272	0.430	0.400	1	0.762 (0.328, 1.771)	0.527
Male	12.60 (8.784-16.41)								
Female	6.300 (0.000-12.56)								
Age		1.680 (0.927-3.044)	0.087	0.717	0.353	4.119	1	2.047 (1.025, 4.090)	0.042
<65	8.733 (1.032-16.43)								
≥65	13.63 (9.292-17.97)								
Smoking history		2.406 (1.227-4.718)	0.002	1.228	0.429	8.176	1	3.414 (1.471, 7.920)	0.004
Non-smokers	6.300 (0.323-12.27)								
Smokers	13.80 (5.362-22.30)								
Histology		1.276 (0.690-2.360)	0.437						
ADC	11.86 (7.937-15.80)								
Non-ADC	10.50 (8.280-12.72)								
Histology		1.081 (0.569-2.055)	0.811						
SCC	10.50 (8.369-12.63)								
Non-SCC	11.86 (7.919-15.82)								
Stage		0.447 (0.238-0.842)	0.040	0.331	0.473	0.491	1	1.393 (0.551, 3.519)	0.483
III	28.60 (13.68-25.70)								
IV	10.20 (6.899-13.43)								
Distant metastases		0.446 (0.242-0.823)	0.030						
No	28.60 (13.84-25.05)								
Yes	10.20 (6.975-13.36)								
Tumor size		1.644 (0.916, 2.950)	0.096	0.880	0.349	6.349	1	2.411 (1.216, 4.779)	0.012
<3 cm	14.67 (10.19-19.14)								
≥3 cm	9.133 (6.945-11.32)								
Camrelizumab treatment		1.007 (0.560, 1.812)	0.980						
Monotherapy	10.50 (6.930-14.10)								
Combination	11.87 (6.623-17.11)								
Treatment interval		1.291 (0.720, 2.315)	0.391						
3 weeks	10.43 (6.442-14.42)								
2 weeks	12.20 (9.084-15.32)								
Response to camrelizumab		0.355 (0.200-0.628)	0.002	1.318	0.418	9.960	1	3.735 (1.648, 8.465)	0.002
SD+PD	9.100 (4.903-12.56)								
CR+PR	25.40 (8.347-42.45)								
Response to camrelizumab		6.141 (3.079-12.25)	<0.0001						
CR+PR+SD PD	22.00 (19.95-14.13) 4.000 (1.227-6.839)								

ADC, Adenocarcimona; CR, complete response; ECOG, Eastern Cooperation of Oncology Group; Non-ADC, non-adenocarcinoma; Non-SCC, non-squamous lung cancer; PD, progression disease; PR, partial response; SCC, squamous cell lung cancer; SD, stable disease.

**Figure 4:**
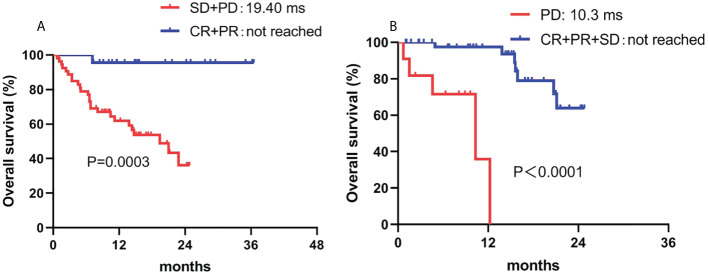
Correlation between OS and **(A)** ORR and **(B)** DCR. ORR, overall response rate; DCR, disease control rate.

The response to camrelizumab was the only independent prognostic factor for OS in both the univariate and multivariate analyses. Patients who achieved an objective response had superior OS compared to those who had stable disease or PD (not reached vs. 19.4 months, *P* = 0.007). Although sex showed a tendency for significance in the univariate analysis, no significant difference was observed in the multivariate analysis (**Table 5**; **Figures 4**).

**Table 5:** The univariate and multivariate analyses of overall survival.

	Univariate analyses Multivariate analyses								
	Median OS (95% CI)	HR(95% CI)	*P* value	β	S.E.	Wald	df	HR(95% CI)	*P* value
Gender(Female *vs.* Male)		2.046(0.909, 4.604)	0.084	0.275	0.439	0.394	1	1.317(0.557, 3.112)	0.530
Male	*26.54(22.61-30.47)								
Female	14.67(7.628-21.70)								
Age		1.368(0.574, 3.260)	0.480						
<65	*26.34(20.43-32.24)								
≥65	* 24.11(19.73-28.48)								
Smoking history		1.312(0.582, 2.956)	0.512						
Non-smokers	21.00(13.91-28.01)								
Smokers	*24.88(20.96-28.80)								
Histology		1.307(0.567, 3.012)	0.530						
ADC	*24.01(19.57-28.45)								
Non-ADC	*25.94(20.76-31.13)								
Histology		1.186(0.497, 2.826)	0.701						
SCC	*25.49(19.76-31.21)								
Non-SCC	*24.44(20.21-28.68)								
Stage		1.736(0.596, 5.057)	0.312						
III	*23.30(18.82-27.79)								
IV	* 23.96(19.91-27.99)								
Distant metastases		2.037(0.700, 5.928)	0.192	0.392	0.576	0.465	1	1.481(0.479, 4.577)	0.495
Yes	* 23.56(19.44-27.68)								
No	* 23.77(19.62-27.91)								
Tumor size		1.054(0.487, 2.285)	0.893						
<3 cm	* 24.74(19.94-29.54)								
≥3 cm	* 25.07(19.94-30.19)								
Camrelizumab treatment		1.184(0.546, 2.565)	0.669						
Monotherapy	* 24.93(20.20-29.66)								
Combination	* 24.15(18.74-29.57)								
Treatment interval		1.096(0.502, 2.389)	0.818						
3 weeks	* 25.32(20.20-30.43)								
2 weeks	* 24.48(19.67-29.28)								
Response to camrelizumab		15.10(6.830, 33.38)	0.0003	2.651	1.026	6.674	1	14.17(1.896, 105.8)	0.010
CR+PR	*not reached								
SD+PD	*19.40(11.17-27.63)								
Response to camrelizumab		9.850(1.035, 93.78)	<0.0001						
CR+PR+SD	* not reached								
PD	*10.3(3.454-24.15)								

*Mean overall survival. ADC, Adenocarcimona; CR, complete response; ECOG:Eastern Cooperation of Oncology Group;Non-ADC, Non-adenocarcinoma; Non-SCC: Non-squamous lung cancer; PD, Progression disease; PR, Partial response; SCC: Squamous cell lung cancer; SD, Stable disease.

### 3.4 Safety of MWA plus camrelizumab

No peri-procedural death from ablation was observed. Complications were observed in 33 patients (42.9%). Major complications included pneumothorax (14, 18.2%), pleural effusion (9, 11.7%), pneumonia (4, 5.2%), bronchopleural fistula (2, 2.6%), and hemoptysis (1, 1.3%), which were all treated by chest tube insertion, anti-infection therapy, blood transfusion, or symptomatic treatment. Minor complications of pneumothorax, pleural effusion, and hemorrhage were identified in 10, 9, and 1 patient(s), respectively (**Table 6**).

**Table 6:** The complications of microwave ablation.

	N=77	Percent(%)
Complications	33	42.9
Major complications		
Pneumothorax	14	18.2
Pleural effusion	9	11.7
Pneumonia	4	5.2
Bronchopleural fistula	2	2.6
Hemorrhage	1	1.3
Minor complications		
Pneumothorax	10	13.0
Pleural effusion	9	11.7
Hemorrhage	1	1.3

AEs were identified in 40 patients (51.9%), and serious AEs were observed in 12 patients (15.6%). Common AEs of camrelizumab included reactive capillary endothelial proliferation (22, 28.6%), fatigue (9, 11.7%), pneumonia (7, 9.1%), edema (5, 6.5%), and fever (5, 6.5%). Grade 3 or higher AEs developed in a corresponding 10.4%, 6.5%, 5.2%, 2.6%, and 2.6% of patients, respectively. Two patients died of serious immune-associated pneumonia (**Table 7**).

**Table 7:** The adverse events of camrelizumab.

Adverse events	All (N)	Percent (%)	≥Grade 3 (N)	Percent (%)
Total	40	51.9	12	15.6
Reactive capillar hemangiomas	22	28.6	8	10.4
Fatigue	9	11.7	5	6.5
Pneumonia	7	9.1	4	5.2
Edema	5	6.5	2	2.6
Fever	5	6.5	2	2.6
Hemorrhagic tendency	3	3.9	0	0.0
Alanine transaminase elavation	2	2.6	2	2.6
Acute myocardial infarction	2	2.6	2	2.6
Autoallergic	2	2.6	1	1.3
Leukopenia	1	1.3	1	1.3
Neutropenia	1	1.3	1	1.3

## 4 Discussion

In this study, we explored the use of a combination of MWA and camrelizumab for NSCLC treatment. A total of 77 patients were enrolled. The technical success and technique efficacy were 100% and 97.4%, respectively. The ORR was 29.9%. The PFS and OS were 11.8 months and not reached, respectively. No ablation-associated deaths occurred. Complications were observed in 33 patients (42.9%), and grade 3 or higher AEs of camrelizumab were identified in 12 (15.6%) patients. MWA plus camrelizumab monotherapy or combination therapy in NSCLC is an effective and safe treatment regimen.

Camrelizumab was approved as the first-line treatment for advanced NSCLC based on the results of two multicenter, randomized phase III clinical trials (23, 24). CameL was the first phase III clinical trial of camrelizumab as a first-line regimen for advanced, non-squamous NSCLC. PFS was significantly prolonged with camrelizumab plus chemotherapy compared with chemotherapy alone (11.3 months vs. 8.3 months; hazard ratio 0.60; P = 0.0001) (26). The other phase III clinical trial, CameL-sq, compared the combination of camrelizumab and chemotherapy with placebo plus chemotherapy as first-line treatment for advanced squamous cell lung cancer. The camrelizumab plus chemotherapy arm achieved a PFS of 8.5 months, and the OS was not reached, whereas the PFS and OS of the placebo plus chemotherapy arm were 4.9 months and 14.5 months, respectively (27). Camrelizumab is usually combined with anti-vascular endothelial growth factor targeted therapy. In patients receiving camrelizumab in combination with apatinib as subsequent treatment, the median PFS is 5.7 months, and the OS is 15.5 months (28). Further, in patients receiving camrelizumab and anlotinib, the ORR is 28.4%, and the PFS and OS are 6.9 months and 14.5 months, respectively. These results verify that camrelizumab could be used for advanced NSCLC (29).

According to the National Comprehensive Cancer Network guidelines, thermal ablation, including MWA, could be an alternative treatment for patients with contraindications to radical surgery (30). Moreover, MWA could be a treatment option for NSCLC patients with oligometastases or oligoprogression (31, 32). Several studies have explored the application of MWA in advanced NSCLC and have verified that MWA can prolong survival and improve prognosis (18–20). Our previous study explored the combination of MWA plus camrelizumab in 21 patients; the ORR was 33.3%, and the median PFS was 5.1 months (21). In this large-sample, retrospective study, the ORR was similar to that in our previous report. The PFS was significantly prolonged by this treatment and was similar to that of combination camrelizumab and platinum-doublet chemotherapy, indicating that camrelizumab plus MWA may be an alternative combination regimen.

With regard to the efficacy of MWA, previous reports have shown a technique efficacy of nearly 100%; however, we achieved a technique efficacy of only 97.4%. This was because two patients with tumors ≥5 cm failed to achieve complete ablation. Several studies have shown that the rate of completed ablation decreases for tumors ≥3 cm (33, 34). According to previous reports, major complications of MWA include pneumothorax, pleural effusion, pneumonia, bronchopleural fistula, and hemoptysis, which is in accordance with our study results (35, 36). Pneumothorax and pleural effusion were the most common complications of MWA observed in this study. No ablation-associated deaths were observed, indicating that ablation is a safe treatment method.

Patients who underwent camrelizumab combination treatment also had AEs. The common AEs of combination camrelizumab and chemotherapy include decreased neutrophil count, decreased white blood cell count, anemia, and decreased platelet count (26, 27). For combination targeted therapy and camrelizumab, hypertension, palmar-plantar erythrodysesthesia syndrome, increased gamma-glutamyl transferase, transaminitis, and proteinuria are common AEs (28, 29). In this study, the most common AEs were reactive capillary endothelial proliferation, fatigue, pneumonia, edema, and fever. Moreover, grade 3 or higher AEs were observed in 15.6% of patients. These results proved that camrelizumab monotherapy or combination therapy is safe for advanced NSCLC.

The combination of MWA and immunotherapy had similar ORR with other combinations such as immunotherapy with chemotherapy and immunotherapy with targeted therapy. Meanwhile, we could identify that the ORR of MWA plus immunotherapy alone or combination therapy had a superior response compared to previous reports of immunotherapy alone. Several potential mechanisms may have led to this advantage, one was that MWA could have increased the probability of CD8+ tumor infiltrating lymphocytes (TIL) and Natural killer (NK) cells, and the other was that MWA could have increased the peripheral IL-2 and IFN-γ (37). Both CD8+ TIL and cell factors could have enhanced the immune effects, which may have exerted a synergic effect with immunotherapy. Moreover, PD-1 blockade boosts radiofrequency ablation-elicited adaptive immune responses by update PD-L1 expression in colorectal liver metastases (38). PD-L1 expression predicts the superior response to immunotherapy (38). Integrating locoregional therapies such as radiofrequency ablation into anti-PD-1/PD-L1 agent regimens may help release tumor-associated antigens and mediate T-cell immune enhancement and, in the long run, improve the ongoing efficacy of checkpoint inhibitors (39).

Compared with our previous study, the median PFS was prolonged in this study. Three major factors led to the differences. First, 41.6% of patients received immunotherapy combination treatment in this study, whereas all the patients underwent only immunotherapy in the previous study. Second, the median follow-up period reached 13.7 months in this study, but the median follow-up period then was just 6 months. Third, 77 patients were enrolled in this study, but only 21 patients were enrolled in the previous study.

This study had two major limitations. First, patients treated with either camrelizumab monotherapy or combination therapy were included in the study, and it is likely that combination therapy had an effect on the ORR or survival. Second, for patients who received camrelizumab combination therapy, the regimens included chemotherapy, targeted therapy, and chemotherapy plus targeted therapy.

In conclusion, MWA plus camrelizumab monotherapy or combination therapy is an effective and safe treatment regimen for advanced NSCLC. Further prospective, randomized, controlled studies are warranted.

## Data availability statement

The raw data supporting the conclusions of this article will be made available by the authors, without undue reservation.

## Ethics statement

The studies involving human participants were reviewed and approved by The ethics committees of the First Affiliated Hospital of Shandong First Medical University and Shandong Provincial Hospital affiliated to Shandong First Medical University approved this study. The patients/participants provided their written informed consent to participate in this study. Written informed consent was obtained from the individual(s) for the publication of any potentially identifiable images or data included in this article.

## Author contributions

YHH, JW, YTH: Conceptualization, data curation, methodology, and writing—original draft. YTH, ZW, XYa, XYe: Data curation, methodology, resources, and writing—final draft. GW, HC, MW: Writing— review and editing. All authors contributed to the article and approved the submitted version.

## Funding

The study was approved by the National Natural Science Foundation (No. 81901851 and No. 82072028).

## Conflict of interest

The authors declare that the research was conducted in the absence of any commercial or financial relationships that could be construed as a potential conflict of interest.

## Publisher’s note

All claims expressed in this article are solely those of the authors and do not necessarily represent those of their affiliated organizations, or those of the publisher, the editors and the reviewers. Any product that may be evaluated in this article, or claim that may be made by its manufacturer, is not guaranteed or endorsed by the publisher.

## Abbreviations

NSCLC, non-small cell lung cancer; PD-1, programmed death-1; PD-L1, programmed death ligand-1; AE, adverse event; MWA, microwave ablation; ORR, overall response rate; CT, computed tomography; CR, complete response; PR, partial response; PD, progressive disease; PFS, progression-free survival; OS, overall survival.
